# Evidence-based clinical practice guidelines for polycystic kidney disease 2014

**DOI:** 10.1007/s10157-015-1219-7

**Published:** 2016-04-20

**Authors:** Shigeo Horie, Toshio Mochizuki, Satoru Muto, Kazushige Hanaoka, Yoshimitsu Fukushima, Ichiei Narita, Kikuo Nutahara, Ken Tsuchiya, Kazuhiko Tsuruya, Koichi Kamura, Saori Nishio, Tatsuya Suwabe, Yoshifumi Ubara, Eiji Ishimura, Koichi Nakanishi, Keiichi Furukawa, Kenjiro Kimura, Seiichi Matsuo

**Affiliations:** Juntendo University Graduate School of Medicine, Tokyo, Japan; Tokyo Women’s Medical University, Tokyo, Japan; Teikyo University School of Medicine, Tokyo, Japan; Department of Internal Medicine, Jikei University School of Medicine, Tokyo, Japan; Shinshu University School of Medicine, Matsumoto, Japan; Niigata University Graduate School of Medical and Dental Sciences, Niigata, Japan; Kyorin University, Tokyo, Japan; Graduate School of Medical Sciences, Kyushu University, Fukuoka, Japan; National Hospital Organization Chiba-East Hospital, Chiba, Japan; Hokkaido University Graduate School of Medicine, Sapporo, Japan; Toranomon Hospital, Tokyo, Japan; Osaka City University Graduate School of Medicine, Osaka, Japan; Wakayama Medical University, Wakayama, Japan; St. Luke’s International Hospital, Tokyo, Japan; St. Marianna University School of Medicine, Kawasaki, Japan; Nagoya University Graduate School of Medicine, Nagoya, Japan

## Preface

### 1. Origins of the guidelines

Autosomal dominant polycystic kidney disease (ADPKD) is the most common hereditary kidney disease, with approximately half of the patients experiencing end-stage renal disease by age 60. Bilateral cysts progressively proliferate and enlarge, even as complications such as hypertension, hepatic cysts, and intracranial aneurysms lead to more lethal events such as cyst infections and ruptured intracranial aneurysms prior to end-stage renal disease. Early-stage diagnosis and intervention are recognized as being vital. Autosomal recessive polycystic kidney disease (ARPKD) is estimated to occur in 1 in 10,000–40,000 births, with symptoms present neonatally. Due to early detection and management as well as improvements in end-stage renal disease treatment, long-term survival is currently possible in patients other than neonates with severe pulmonary hypoplasia.

In Japan, Clinical Guidelines for Polycystic Kidney Disease in 1995 was published by the Progressive Renal Diseases Research, Research on intractable disease, from the Ministry of Health, Labour and Welfare of Japan, followed by a 2002 revision, the ADPKD Guidelines (second edition). Both serve as protocols for daily treatment of ADPKD in Japan. However, subsequent advancements in PKD expertise led to the 2010 Clinical Guidelines for Polycystic Kidney Disease, which were aimed at physicians and other health practitioners. These events provided the backdrop for the 2014 Clinical Practice Guidelines for Polycystic Kidney Disease, which were drawn up to answer the questions of physicians specializing in renal care.

### 2. The intended purpose, anticipated users, and predicted social significance of the guidelines

The 2014 Clinical Practice Guidelines for Polycystic Kidney Disease were drawn up to assist renal care specialists with daily diagnosis and treatment of ADPKD and ARPKD. These Guidelines offer descriptive and exhaustive coverage of PKD diagnosis and definition, epidemiology, and screening. Moreover, routine treatment by renal specialists is addressed through clinical questions (CQs) and responses. Each response is accompanied by a recommendation grade reflecting the level of evidence the response embodies. Our objective is to convey standardized care through specific responses to renal specialists’ questions, thereby supporting these professionals as they face daily clinical decisions. We anticipate that general practitioners using the current Guidelines along with the 2010 Clinical Guidelines for Polycystic Kidney Disease will deepen their understanding of PKD and liaise more smoothly with renal specialists. The Guidelines should also enhance patients’ understanding of the disease and serve as a reference in answering their questions concerning current treatments.

Professional literature and international conferences afford renal specialists fragmented bits of information about the field, while the specialists are expected to have an integrated understanding of the expertise level and medical environment in Japan, and to provide optimal care for each patient. The current Guidelines incorporate the wisdom of experienced specialists, offering not only evidence, but also practical and standardized views communicated to readers through the CQ responses. However, the degree to which information in these Guidelines may be applied to individual patients requires the judgment of each specialist. Patients do not expect uniform, rigid treatment. Indeed, these Guidelines are not intended to restrict the treatment options available to renal specialists, but rather to facilitate treatment based on their own flexible insights and expert understanding. We must also clarify that the Guidelines are not designed for use in resolving medical practice disputes or as evaluation criteria in malpractice lawsuits.

### 3. Patients within the scope of the guidelines

These Guidelines apply to any and all PKD patients. Section “Origins of the guidelines”, “The intended purpose, anticipated users, and predicted social significance of the guidelines” “Patients within the scope of the guidelines” and “Preparation procedure” address ADPKD, whereas Sections “Contents of the guideline”, “Evidence levels and recommendation grades”, “Issues on the preparation of this guideline”, “Financial sources and conflict of interest” and “Publication and future revisions” cover ARPKD. The Guidelines provide an outline and definition (“Origins of the guidelines” and “Contents of the guideline” section) for each of the two diseases, along with information on diagnosis (“The intended purpose, anticipated users, and predicted social significance of the guidelines” and “Evidence levels and recommendation grades” section), epidemiology (“Patients within the scope of the guidelines” and “Issues on the preparation of this guideline” section), and treatment (“Preparation procedure” and “Publication and future revisions” section). Each section applies to patients regardless of gender or age. However, the Guidelines do not generally take pregnancy into account.

### 4. Preparation procedure

Guidelines on four diseases (IgA nephropathy, nephrotic syndrome, RPGN, and polycystic kidney disease [PKD]) were created simultaneously by a research group on progressive kidney disorders (led by Seiichi Matsuo) funded by the Ministry of Health, Labour, and Welfare’s research project for overcoming intractable diseases. All of these guidelines have the same chapter structure. PKD is a genetic disease, so Shinshu University professor Yoshimitsu Fukushima assisted by serving on the drafting committee as a representative of the Japan Society of Human Genetics. Keiichi Furukawa of the Division of Infectious Diseases in the Department of Internal Medicine at St. Luke’s International Hospital provided assistance regarding cyst infections. We would like to take this opportunity to thank these two physicians for their generous help.

Seventeen CQ were created based on questions the committee members had from actual clinical practice. These guidelines were completed owing to the dedication and effort of the physicians who served on the PKD working group. We thank them again for their efforts (shown separately: 2014 evidence-based PKD clinical guidelines committee).

### 5. Contents of the guideline

Guidelines on four diseases (IgA nephropathy, nephrotic syndrome, RPGN, and PKD) with the same format and structure were drafted by a research group on progressive kidney disorders (led by Seiichi Matsuo) funded by the Ministry of Health, Labour, and Welfare’s research project for overcoming intractable diseases. As described earlier, the first half (chapters 1–4) addresses ADPKD and the second half (chapters 5–8) addresses ARPKD.

### 6. Evidence levels and recommendation grades

Evidence was classified into six levels based on the study design, and was arranged roughly from the most reliable study type (Level 1) to the least reliable (Level 6). These levels do not necessarily represent rigorous scientific standards; they are intended for use as a convenient reference for quickly assessing the significance of various clinical data during the physician’s decision-making process.

[Evidence Levels]

- Level 1: Systematic review/meta-analysis.
- Level 2: At least one randomized controlled trial (RCT).
- Level 3: A non-RCT.
- Level 4: An analytical epidemiologic study (cohort study or case-control study) or a single-arm intervention study (no controls).
- Level 5: A descriptive study (case report or case series).
- Level 6: Opinion of an expert committee or an individual expert, which is not based on patient data.

However, for a systematic review/meta-analysis, the evidence level was decided based on the designs of the underlying studies. If the underlying study designs were mixed, the lowest level underlying study was used to determine the overall evidence level. For example, a meta-analysis of cohort studies would be Level 4, but the same Level 4 would also be assigned to a meta-analysis including both RCTs and cohort studies.

In addition, a decision based on committee consensus was that all sub-analyses and post-hoc analyses of RCTs should be categorized at evidence Level 4. Accordingly, it was decided that the evidence level of findings representing the primary endpoints of an RCT would be Level 2, but the evidence level of findings determined via a sub analysis or post-hoc analysis of that RCT would be Level 4.

When a statement related to a certain treatment was presented, consideration was given to the level of the evidence serving as the basis of that statement, and a recommendation grade was assigned as outlined below:

[Recommendation grades]

- Grade A: Strongly recommended because the scientific basis is strong.
- Grade B: Recommended because there is some scientific basis.
- Grade C1: Recommended despite having only a weak scientific basis.
- Grade C2: Not recommended because there is only a weak scientific basis.
- Grade D: Not recommended because scientific evidence shows the treatment to be ineffective or harmful.

If we found only a weak scientific basis for a certain statement concerning a treatment, the members of the committee discussed the matter and decided on C1 or C2 for the recommendation grade. Thus, discrimination between C1 and C2 statements was based on expert consensus.

### 7. Issues on the preparation of this guideline

#### (1) Paucity of evidence

Little evidence exists for PKD, and only few large clinical studies have been performed globally, apart from a small number in the United States and Europe. For the most part, little evidence substantiates the recommendations in the CQ. In particular, almost no evidence comes from Japan. Whether the results of clinical research from the West can be applied as is to Japan is a question that deserves careful consideration. In creating these guidelines, we strove to ensure that they would not deviate greatly from the clinical practice in Japan.

#### (2) Issues on medical resources

In general, the clinical guideline must consider medical resources associated with recommended statements. However, the current guideline did not discuss issues on medical cost; thus medical financial problems did not affect the contents of our guideline. In the next guideline, this point may be included.

#### (3) Guideline reflecting the opinions of patients

During the preparation processes of the clinical guideline, we needed to introduce the opinions of patients. However, this time, we unfortunately could not include the opinions of patients. We should refer to the opinions of patients in the next guideline, particularly in the case that the guideline is used for patients.

### 8. Financial sources and conflict of interest

The funds used in creating the guidelines were provided by a research group on progressive kidney disorders funded by the Ministry of Health, Labour, and Welfare’s research project for overcoming intractable diseases. These funds were used to pay for transportation to and from meetings, to rent space for meetings, and for box lunches and snacks. The committee members received no compensation. Everyone involved in creating the guidelines (including referees) submitted conflict-of-interest statements based on academic society rules, which are managed by JSN. Opinions were sought from multiple referees and related academic societies to prevent the guidelines from being influenced by any conflicts of interest. Drafts were shown to the society members, and revisions were made based on their opinions (public comments).

### 9. Publication and future revisions

The Guidelines were published in the Japanese-language journal of the Japanese Society of Nephrology and concurrently released as a Japanese-language book (by Tokyo Igakusha, Tokyo). The Guidelines were also uploaded to the homepage of the Japanese Society of Nephrology.

At present, CKD-related evidence is being rapidly accumulated, and this new evidence will necessitate the preparation of an updated version of the Guidelines in 3–5 years. A certain degree of turnover in the membership of the revision committee will be required in order to ensure the impartiality of the Guidelines.

## 1. Disease concept and definition of ADPKD

ADPKD is the most common hereditary cystic kidney disease. ADPKD is characterized by the progressive development of fluid-filled cysts derived from renal tubular epithelial cells and the development of disorders in several organs. Bilateral renal cysts enlarge progressively, gradually compromising renal function, and finally, end-stage renal disease (ESRD) requiring renal replacement therapy occurs in approximately 50 % of patients by the age of 60 years.

The pattern of transmission in ADPKD is autosomal dominant inheritance. A male or female with a mutant allele develops the disease. In case that both parents are unaffected, disease in the offspring results from new mutation.

ADPKD is caused by a germ line mutation in *PKD1* (16p13.3)(85 % of cases) or *PKD2* (4q21)(15 % of cases).

## 2. Diagnosis of ADPKD: symptoms and laboratory findings

### (1) Algorithm

**Fig. 1:**
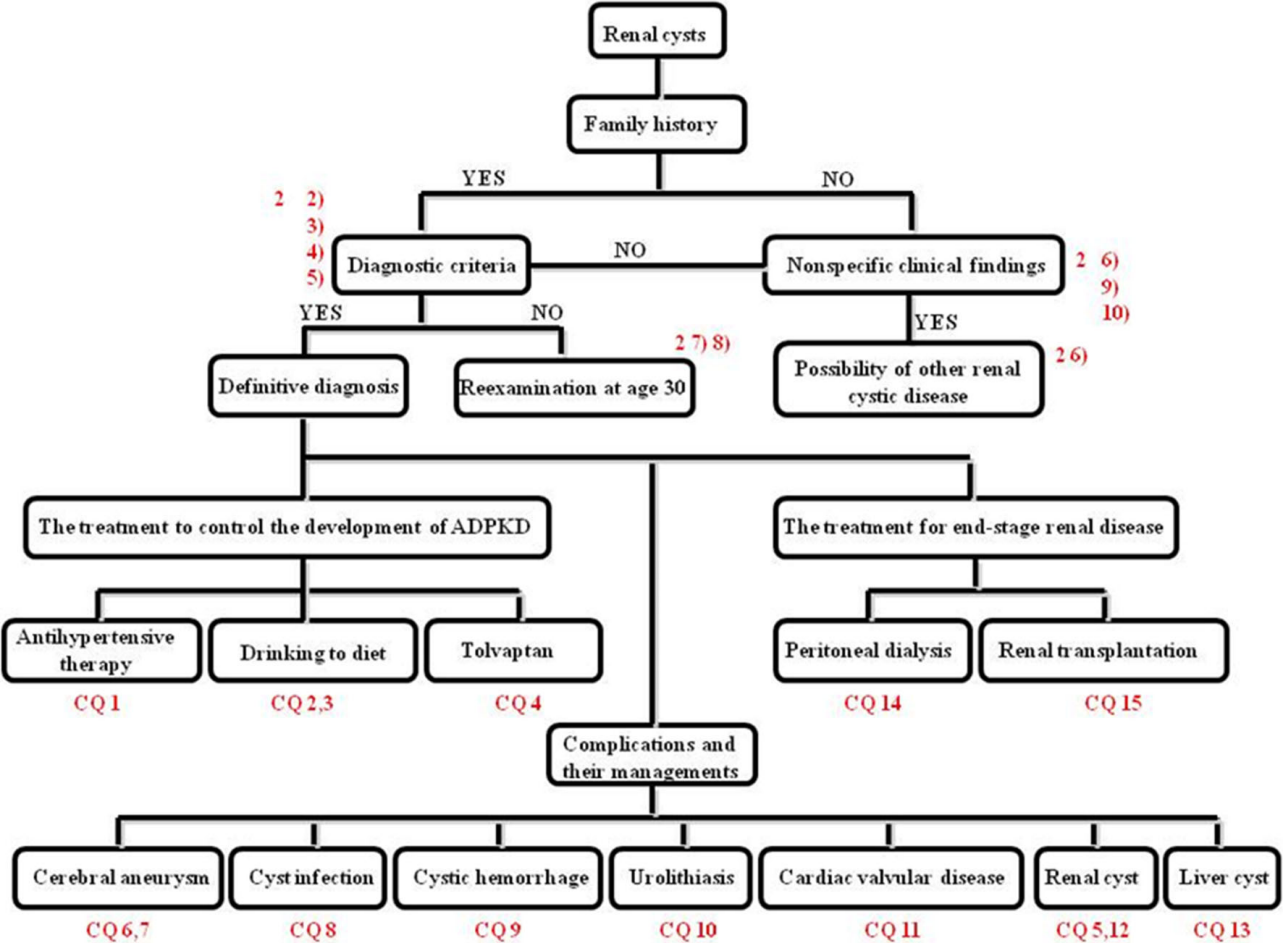
The algorithm for diagnosis of ADPKD patients

The diagnostic algorithm for ADPKD is depicted in the Fig. 1. Family history, while important in ADPKD diagnosis, often cannot be assessed. Moreover, even in the absence of family history, it is important to remain alert to newly reported mutations in *PKD1*/*PKD2* genes responsible for disease onset. It can be difficult to detect cysts meeting diagnostic criteria in younger patients, requiring reexamination. Clinical questions (CQs) are appended to these guidelines as a reference in following the algorithm and determining treatment and other medical care once a definitive diagnosis has been made.

### (2) Diagnostic criteria

Table 1 presents the diagnostic criteria of ADPKD (*ADPKD Diagnostic Guidelines, Second Edition*, published by a Grant-in-Aid for Progressive Renal Diseases Research, Ministry of Health, Labour and Welfare of Japan). Confirmation or nonconfirmation of family history determines one of two possible protocols, each requiring its own distinctive cyst assessment based not only on ultrasonography (US) but also on computed tomography (CT) and magnetic resonance imaging (MRI). In most cases, cysts manifest bilaterally and diagnosis is uncomplicated; in the remaining cases, diagnosis should be carefully performed in accordance with the diagnostic criteria noted herein.

**Table 1:** The diagnostic criteria of ADPKD (*ADPKD Diagnostic Guidelines, Second Edition*, published by a Grant-in-Aid for Progressive Renal Diseases Research, Research on intractable disease, from the Ministry of Health, Labour and Welfare of Japan)

(1) Confirmation of family history
(a) Three or more bilaterally-manifested cysts confirmed with ultrasonography
(b) Five or more bilaterally-manifested cysts confirmed with CT and MRI imaging
2. Non-confirmation of family history
(a) Patients 15-years old or younger: three or more bilaterally-manifested cysts confirmed with either CT and MRI imaging or ultrasonography
(b) Patients 16-years old or older: five or more bilaterally-manifested cysts confirmed with either CT and MRI imaging or ultrasonography
Diseases to be excluded
(1) Multiple simple renal cyst
(2) Renal tubular acidosis
(3) Multicystic kidney (multicystic dysplastic kidney)
(4) Multilocular cysts of the kidney
(5) Medullary cystic disease of the kidney (juvenile nephronophthisis)
(6) Acquired cystic disease of the kidney
(7) Autosomal recessive polycystic kidney disease

### (3) Comparison of diagnostic criteria between Japan and other countries

Following Bear’s diagnostic criteria in 1984, numerous other versions have been reported, each with its own emphasis on, for example, age classification or cyst assessment through imaging. Ravine’s criteria, which were utilized for some time, were the first guidelines reflecting age as a factor. However, Ravine only incorporated *PKD1* family history. Although *PKD1* and *PKD2* mutations each result in almost the same clinical manifestation of the disease, *PKD1* progresses to ESRD more rapidly and produces more cysts, leading Pei to incorporate both *PKD1* and *PKD2* families in his diagnostic criteria. Diagnosis in Western countries combining US with genetic testing is highly credible and should serve as a reference, but its applicability to Japanese patients has not yet been demonstrated.

### (4) Testing

ADPKD screening should include family history of renal disease (end-stage and otherwise) and intracranial hemorrhage/cerebrovascular disease; patient history of hypertension, cerebrovascular disease, urinary tract infection, fever, and lower back pain; subjective symptoms such as macroscopic hematuria, lower back and/or flank pain, abdominal distension, headache, edema, and nausea; physical examination to determine blood pressure, abdominal girth, heartbeat, abdominal findings, and edema; blood and urine tests, screening for urinary sediment, proteinuria, and microalbuminuria; estimated glomerular filtration rate (eGFR) and other renal function tests; and screening for intracranial aneurysm through cranial MR angiography. US represents the simplest form of diagnostic imaging for kidney diseases. Other tests to be performed, as appropriate, should include measurement of *N*-acetyl beta-glucosaminidase and urinary beta2 microglobulin values, MRI, and kidney CT imaging.

### (5) Diagnostic imaging

US is the standard screening technique for ADPKD diagnosis and evaluation, but evaluation of kidney size, as opposed to function, is reportedly the better measurement in the evaluation of progression, with CT or MRI recommended for follow-up evaluation. The latter methods surpass US in detecting smaller cysts; MRI can detect cysts with a diameter of 2 mm through T2-weighted imaging. Each diagnostic imaging technique (US, CT, and MRI) plays a role in highlighting the distinctive characteristics of cysts. Diagnostic imaging is also clinically important in terms of disease complications such as cerebral aneurysms. As adverse reactions can occur, careful consideration must be given to the risk–benefit balance before utilizing contrast media. MRA is useful in screening for cerebral aneurysms and is a noninvasive test with the great benefit of not requiring contrast media. If imaging performed after a definitive ADPKD diagnosis is strictly for follow-up observation, a simple CT once every 2–5 years would be adequate if total kidney volume (TKV) is ≤1000 mL. If TKV exceeds 1000 mL, CT once every year or two would be appropriate. For screening purposes, diagnostic imaging at the age of 30 years is recommended.

### (6) Differential diagnosis

A patient’s clinical manifestation and diagnostic imaging should be used to rule out possibilities such as multiple simple renal cysts, acquired cystic kidney disease, and tuberous sclerosis (Table 2). Particular caution is needed when considering tuberous sclerosis, as approximately 30 % of patients with this disease are said to have no typical symptoms other than renal cysts, which are mistakenly attributed to ADPKD. Additional diseases to be ruled out include renal tubular acidosis, multicystic kidney (multicystic dysplastic kidney), multilocular cyst of the kidney, medullary cystic kidney disease, and oral–facial–digital syndrome. As rare diseases are difficult to identify and distinguish during normal medical examinations, despite reports on characteristic indicators other than renal cysts, extra care should be given during differential diagnosis.

**Table 2:** Major non-ADPKD renal cystic diseases

Disease	Cyst proliferation	Cyst distribution/size	Typical life stage for cyst diagnosis	Pathophysiological characteristics
Multiple simple renal cyst	Moderate	Size diversity/non-uniform distribution	All ages	Rare under age 30 years; manifestation increases with age
Acquired cystic disease of the kidney	Moderate to great	Diffusibility	Adulthood	Cyst formation precedes ESRD
Tuberous sclerosis	Moderate to great	Uniform distribution of relatively small (<1 to 2 cm) cysts	All ages	Renal angiomyolipomas, skin lesions, periungual fibromas, retinal hamartomas, and cardiac rhabdomyomas
ARPKD	Great	Diffusibility/small cysts	Birth	Greatly enlarged kidney, congenital hepatic fibrosis

### (7) Genetic diagnosis

ADPKD is an autosomal dominant genetic disease. Responsible genes for ADPKD were already identified. Diagnosis of ADPKD in typical cases is easy by detecting multiple cysts in both kidneys. In Japan, genetic diagnostic tests for ADPKD are only available for basic research but not for clinical practice. Physicians must consider whether samples for genetic testing should be sent to foreign laboratories.

### (8) Diagnostic imaging for infants and young adults

Diagnostic criteria, including imaging, for ADPKD in infants and young adults have not been established. Screening imaging tests are not recommended for nonsymptomatic infants and young adults, even if they are children of ADPKD patients.

### (9) Initial symptoms

Cysts are said to form in utero, with most progressing asymptomatically until the patients are in their 30 or 40 s. Subjective symptoms include abdominal or lower back pain, macroscopic hematuria (including its posttraumatic form caused by sports activities), or abdominal bloating. Acute pain is usually attributable to hemorrhagic cysts, infection, or urinary tract stones. Chronic pain is defined as persistent pain for 4–6-weeks. It occurs in approximately 60 % of ADPKD cases and is usually attributable to cysts. Macroscopic hematuria occurs in approximately 50 % of all cases. Hypertension, diagnosed objectively by physical examination and other methods, is a significant initial symptom (or findings).

### (10) Renal symptoms

Both acute and chronic abdominal and/or flank pain is one of the most prevalent subjective symptoms of ADPKD, whereas many patients do not have any complaint until their third or fourth decade of life. Anorexia, gastrointestinal obstruction, and malnutrition are manifestations of compression of the gastrointestinal tract by the advanced enlargement of the kidney (and/or the liver). Macroscopic hematuria is observed at least once during the entire clinical course in almost 50 % of the patients. Massive proteinuria is rare. The first functional abnormality of the kidney is disturbed concentrating capacity, although it rarely becomes clinically evident unless the patient complains of polydipsia and polyuria. Decrease in GFR usually starts after 40 years of age, and the mean rate of its reduction is 4.4–5.9 mL/(min year).

The factors associated with rapid progression of GFR decline have been reported as follows:

1. Disease-causing gene (worse in cases with *PKD1* mutation than in those with *PKD2* mutation)
2. Hypertension
3. Early development of urinary abnormality (hematuria and proteinuria)
4. Male sex
5. Large size and rapid enlargement of the kidney
6. Left cardiac hypertrophy
7. Proteinuria

## 3. ADPKD: epidemiology and prognosis (prevalence, incidence, renal prognosis, and vital prognosis)

The number of ADPKD patients in Japan who visited hospitals was estimated to be 14,594, yielding an ADPKD prevalence of 116.7 cases per million population at the end of 1994. The total number of ADPKD patients including those who will visit hospitals in the future was estimated to be 31,000. It was suggested that ADPKD affected one individual per 4033 population in Japan. ADPKD was diagnosed in 40 residents of Olmsted County between 1935 and 1980, resulting in an age- and sex-adjusted annual incidence rate of 1.38 case per 100,000 person-years. Approximately 50 % of the patients developed ESRD at the age of 60–69 years. The most common causes of death in ADPKD were infection, sepsis, and cardiac disease (myocardial infarction and congestive heart failure). The survival of ADPKD patients undergoing dialysis surpasses that of general dialysis patients.

## 4. ADPKD: treatment and management of complications

### (1) Treatment to control the development of ADPKD


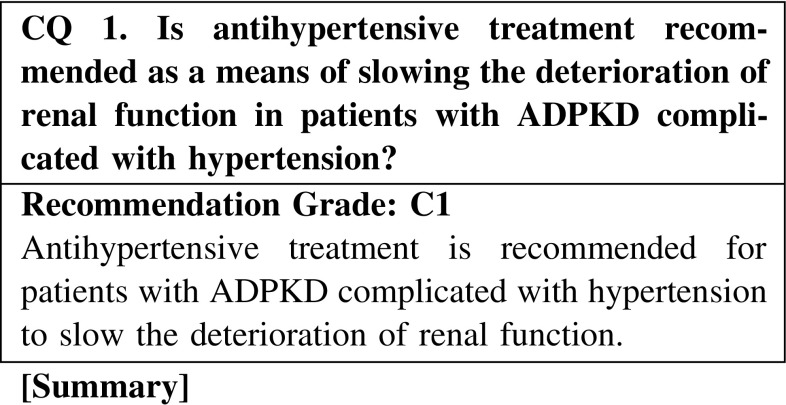


Hypertension in ADPKD is frequent and develops at a young age, in contrast to essential hypertension. In addition, it is often detected when renal function is normal and cysts are still small. Antihypertensive treatment is generally performed. It is thought that antihypertensive treatment may slow the deterioration of renal function in ADPKD with hypertension. However, because the evidence related to the recommended antihypertensive agents and target blood pressure is inconclusive, we recommend that antihypertensive treatment in ADPKD should follow that administered for chronic kidney disease (CKD).
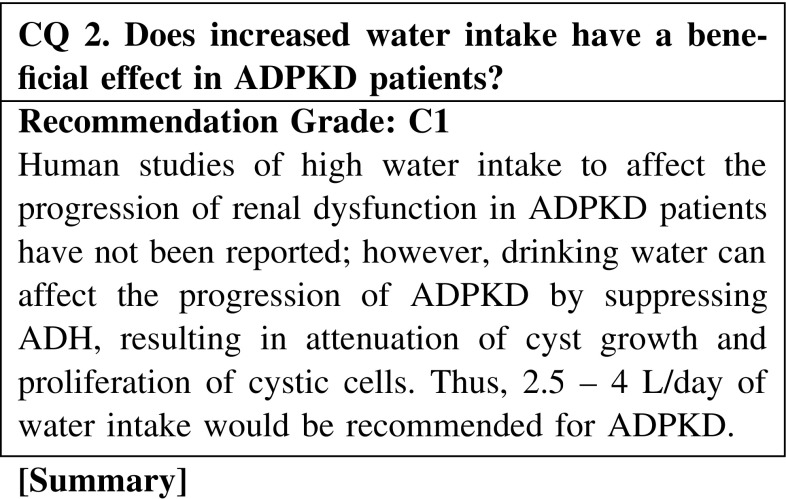


A 3′–5′-cyclic adenosine monophosphate (cAMP)-mediating vasopressin receptor can stimulate cystic cell proliferation and fluid secretion into cysts in ADPKD. Thus, a novel treatment of ADPKD that targets the vasopressin-cAMP axis is currently evaluated and a selective inhibitor of vasopressin two receptor is adopted and examined for its effects on ADPKD. Another way to suppress vasopressin secretion is to increase fluid intake to mediate osmoregulation. Although human studies have not been reported regarding the effect of high water intake on the renal size and function of ADPKD, increasing water intake could be recommended to affect the progression of ADPKD based on the biological properties of the cystic epithelium. A larger human study is needed to clarify the effect of high water intake; patients would be advised to avoid stimulating vasopressin secretion by chronic water depletion.
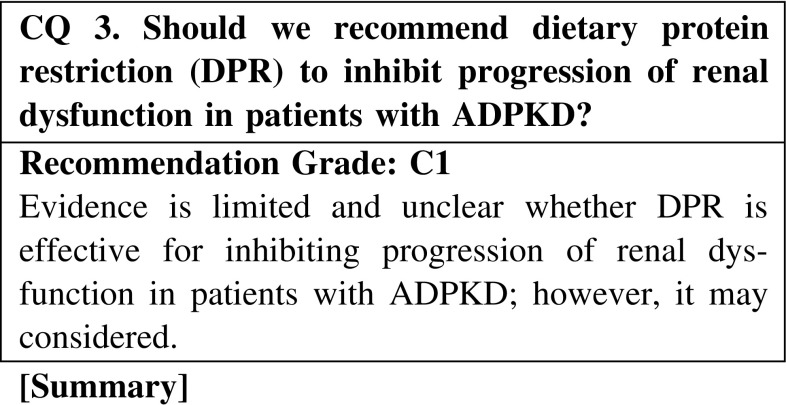


The effect of DPR on ADPKD has been examined by several clinical studies, including small retrospective studies and randomized clinical trials. However, almost all studies have shown no significant effect of DPR on the progression of renal dysfunction. Although a meta-analysis showed the efficacy of DPR in patients with CKD, including ADPKD, the effect in ADPKD patients alone was not evaluated. However, we could not conclude that DPR is ineffective for those patients because of the many limitations of those clinical studies, such as a small sample size, low prevalence of outcome due to a short observation period, and low adherence to DPR. Thus, further evidence is required to answer this question.
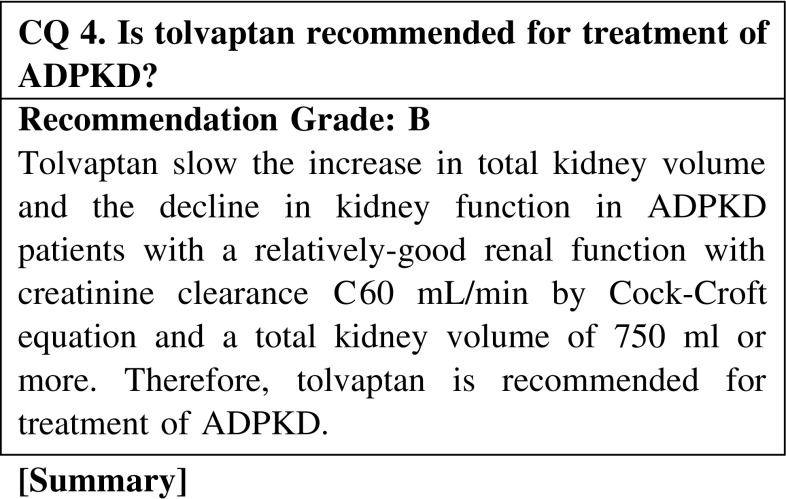


Tolvaptan, a V_2_-receptor antagonist, selectively blocks the binding of vasopressin to the V2-receptors and inhibit production of cAMP. To determine the effect of tolvaptan to suppress the increase in total kidney volume, a phase 3, international multicenter, double-blind, placebo-controlled, 3-year trial (TEMPO3/4) was performed. The results of the trial demonstrated that tolvaptan slowed the increase in total kidney volume and the decline in kidney function in ADPKD patients with a relatively-good renal function with creatinine clearance ≥60 mL/min by Cock-Croft equation and a total kidney volume of 750 mL or more. Due to the lack of other specific and efficacious treatments for ADPKD at present time, with particular attention to serious adverse events such as drug-induced liver injury, tolvaptan is recommend for treatment of ADPKD patients with a relatively-good renal function and a total kidney volume of 750 mL or more. However, the safety of tolvaptan therapy for adult patients with creatinine clearance <60 mL/min or total kidney volume less than 750 mL or children is not established.
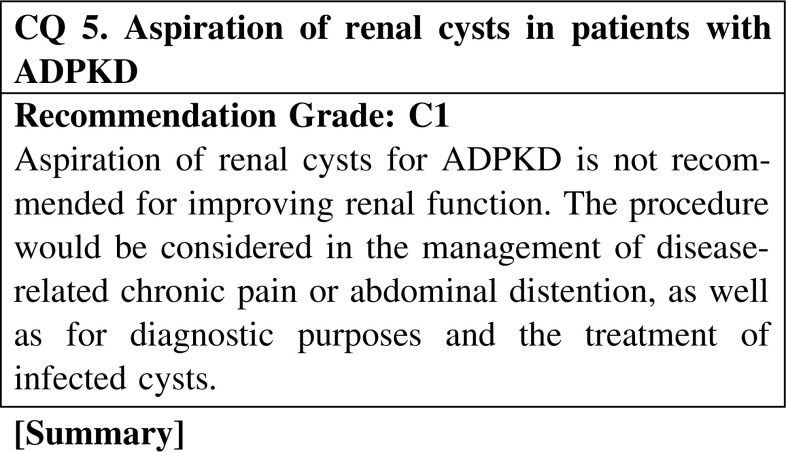


A review of cyst aspiration and surgical cyst decortication for symptomatic ADPKD was performed. The impact of renal cyst aspiration or surgical cyst decortication on renal function and hypertension in patients with ADPKD is controversial, but these procedures are highly effective in the management of disease-related chronic pain. The duration of pain relief is shorter in cyst aspiration than surgical cyst decortication.

The cyst aspiration technique for simple renal cysts can be used for ADPKD. Cyst aspiration followed by instillation of a sclerosing agent (most commonly ethanol) is indicated when the symptoms are caused by one or few dominant or strategically located cysts. Cyst aspiration and sclerosis for multiple cysts need further investigation.

Cyst aspiration for diagnostic purposes and the treatment of infected cysts has been the standard procedure.
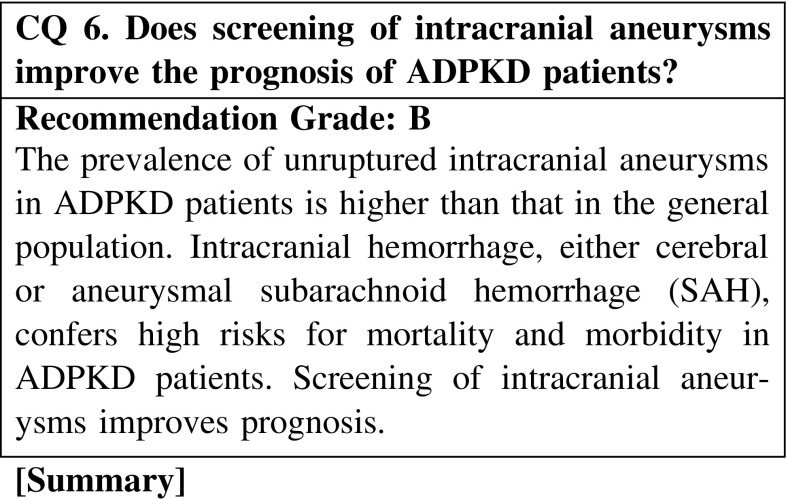


The high incidence of intracranial aneurysms in patients with ADPKD has long been recognized. Rupture of an intracranial aneurysm resulting in SAH is the most devastating extrarenal complications and often results in premature death or disability. The prevalence rate of unruptured intracranial aneurysms in patients with ADPKD is higher than that in people without comorbidity. First-degree relatives (parents, siblings, and children) of patients with subarachnoid hemorrhage have a 3–7 times higher risk for SAH than the general population.

Aneurysm size correlates with the presence of symptoms and the risk of bleeding, and aneurysms may rupture more often and at a younger age than sporadic aneurysms. However, there is no correlation between the risk of rupture and sex, renal function and blood pressure. Hence, it is difficult to predict intracranial aneurysm rupture.

Intracranial hemorrhage, either cerebral hemorrhage or aneurysmal SAH, confers high risks for mortality and morbidity in PKD patients. Screening of intracranial aneurysms improves prognosis.
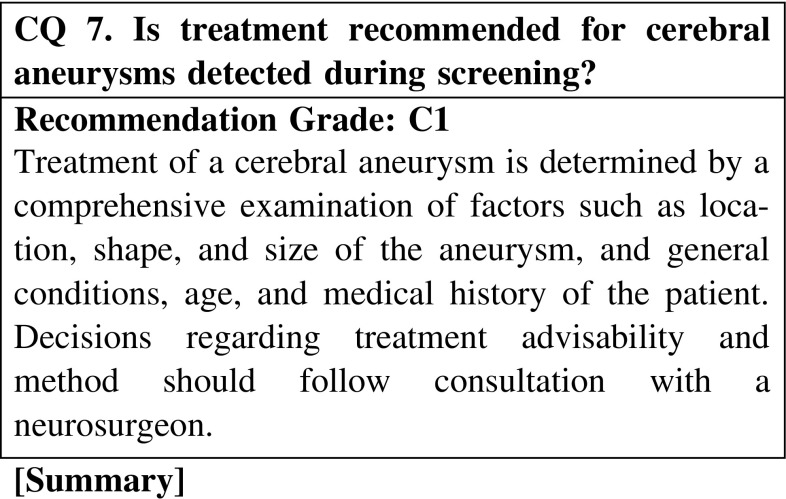


Considering that a ruptured cerebral aneurysm is a life-threatening complication, detection of an unruptured cerebral aneurysm during screening should receive all due attention. However, there is no particular treatment for the latter, which is specific to ADPKD. Detection of a cerebral aneurysm during screening should be followed by careful control of smoking, alcohol consumption, and blood pressure. Treatment of a cerebral aneurysm is surgery, involving a craniotomy and endovascular treatment, with specifics determined following comprehensive investigation of the location, shape, and size of the aneurysm, and general conditions, age, and medical history of the patient. As treatment options have their respective strengths and weaknesses, decisions should follow consultation with a neurosurgeon. If conservative observation is chosen, biannual—or at the very least, annual—monitoring of aneurysm size is recommended.
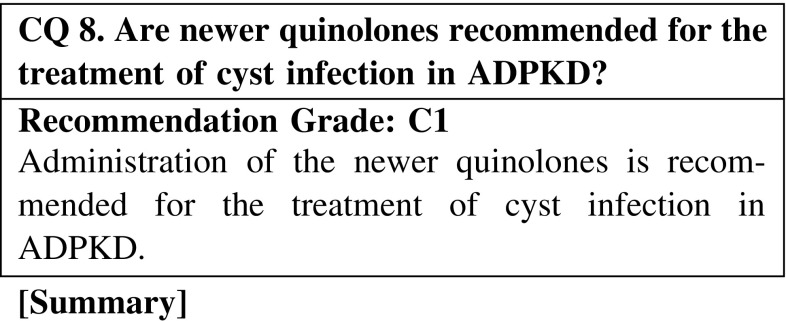


Cyst infection is a frequent and serious complication of ADPKD and is often refractory and difficult to treat. Most causative bacteria originate from the intestine, and many are gram-negative rods. Fluoroquinolones, which have broad effectiveness against gram-negative rods and good penetration of cysts, is recommended for the treatment of infected cysts in ADPKD. Having said this, however, there has not been an adequate level of study to investigate the actual effectiveness of fluoroquinolones for treating cyst infection in ADPKD. Few studies have compared fluoroquinolones with other antibiotics for the treatment of cyst infection in ADPKD.
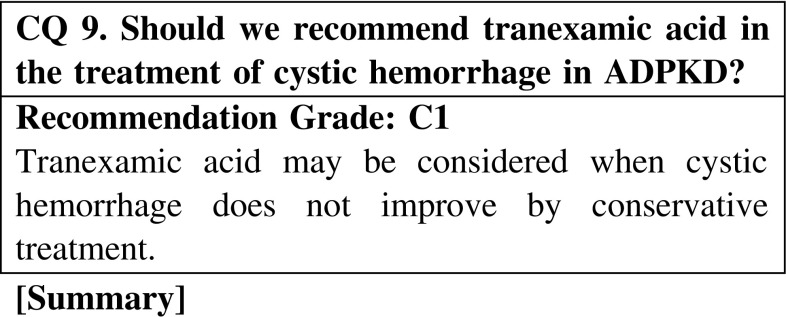


Hematuria is a common problem in patients with polycystic kidney disease. It can be spontaneous or result from trauma, renal calculi, tumor, or infection. These episodes are normally managed with conservative medical treatment and rarely require surgery or embolization. Only a few published studies have investigated the use of tranexamic acid for the treatment of cystic hemorrhage in ADPKD. However, these studies demonstrated that tranexamic acid can be used safely and is effective for selected ADPKD patients with severe or intractable cystic hemorrhage that does not respond to conventional treatment.

Thus, tranexamic acid may be considered when cystic hemorrhage does not improve by conservative treatment.
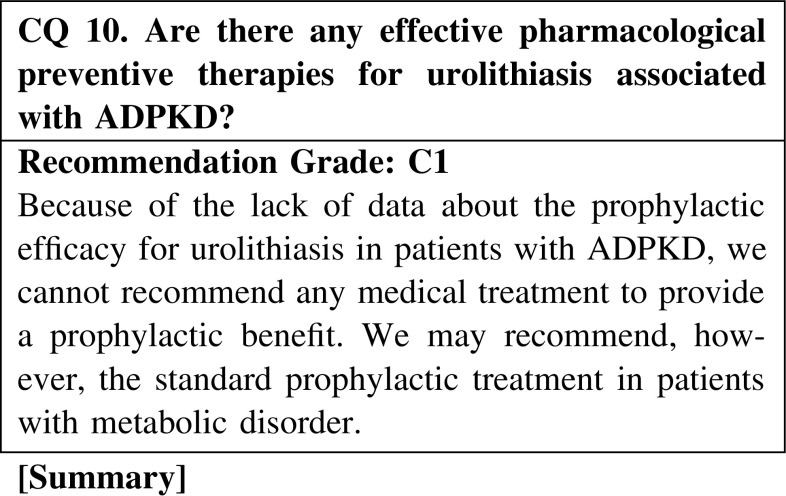


Renal calculi were detected in 21 % of male and 13 % of female patients with ADPKD. Anatomical urinary retention and metabolic disturbance in patients with ADPKD tend to cause development of renal stones. The main component of the stones is uric acid, and the most common metabolic abnormality is hyperoxaluria. Medical preventive treatments are not recommended because of the lack of studies that prove their efficacy. General preventive measures are recommended for fluid intake and diet.
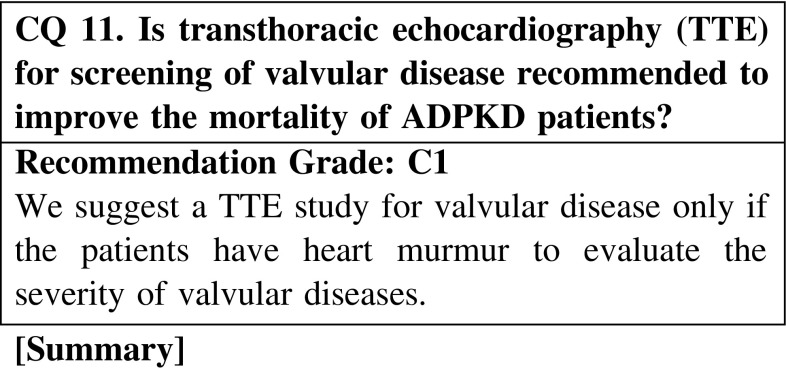


Mitral valve prolapse and mitral regurgitation (MR) are the common cardiac complications in ADPKD. Twenty-one percent of Japanese ADPKD patients have MR. However, solid data on the natural history of valvular disease in ADPKD are currently lacking, and studies with long-term follow-up periods are also very few.

According to the reports regarding non-ADPKD patients, mild or trivial MR carries better prognosis and is thought not to affect the loss of cardiac function and mortality in cardiovascular diseases.

For patients with a heart murmur, it is uncertain whether the disease is mild or severe. TTE might be useful to evaluate indications for surgical treatments and improve the mortality of these ADPKD patients.
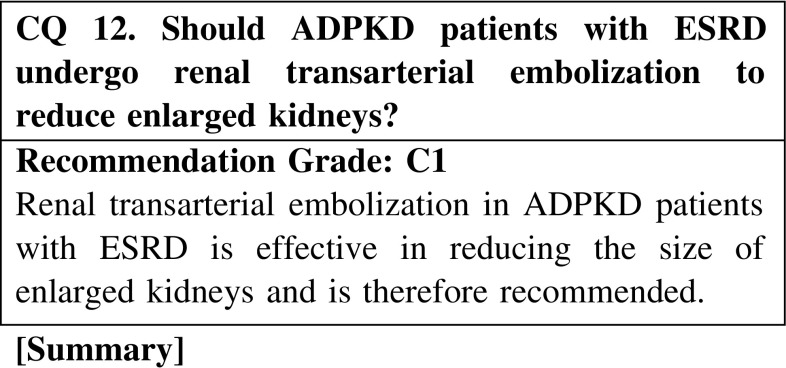


As ADPKD patients age, kidney enlargement becomes increasingly pronounced, with some patients experiencing considerable abdominal bloating. Such patients are unable to eat properly, leading to malnutrition and an overall deterioration of health. However, there is no clear treatment for massively enlarged kidneys. The literature remains sparse on renal transarterial embolization in ADPKD patients with enlarged kidneys, and reports differ as to the embolism type. However, as renal transarterial embolization was demonstrated to reduce kidney swelling in all existing reports, the procedure is believed to be effective for ADPKD patients and is therefore recommended despite the paucity of evidence.
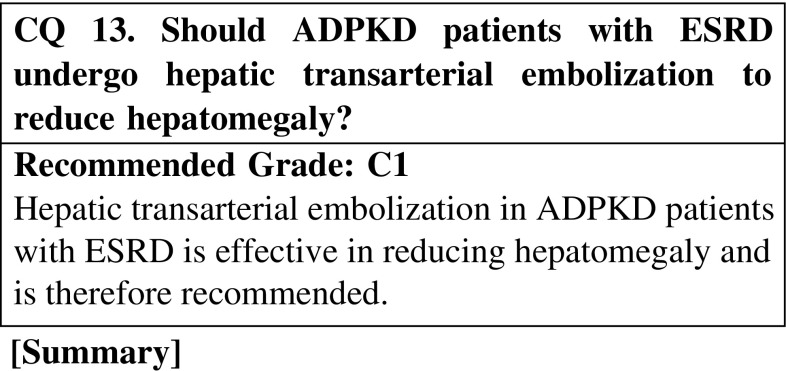


As ADPKD patients age, liver cysts proliferate and hepatomegaly becomes increasingly pronounced, with some patients experiencing extreme abdominal bloating. Such patients are unable to eat properly, leading to malnutrition and an overall deterioration of health. However, there is no clear treatment for a massively enlarged liver. There are limited reports of hepatic transarterial embolization in ADPKD patients with hepatomegaly, but they are individual or collected case reports, as opposed to scientific studies. The evidence presented in these reports is meager, but as there is some suggestion that hepatic transarterial embolization may be effective in ADPKD patients with enlarged livers, the procedure is recommended.
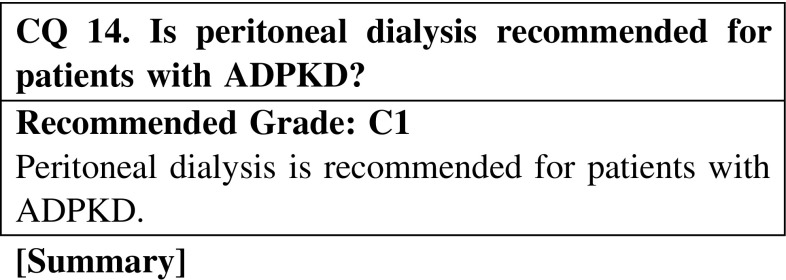


Peritoneal dialysis is not considered appropriate or suitable in ADPKD patients because of the limited peritoneal space due to enlarged kidneys. However, according to the recent European Renal Best Practice Guidelines, initiation of dialysis with peritoneal dialysis should not be considered a contraindication. Which of the two modalities, hemodialysis or peritoneal dialysis, is better for patients’ long survival? Although there have been several studies concerning this question that examined different populations and situations of dialysis patients, there is no definite conclusion or consensus on this matter. The dialysis modalities, hemodialysis or peritoneal dialysis, should be decided by patients themselves according to the suitability of the modality for the patients.
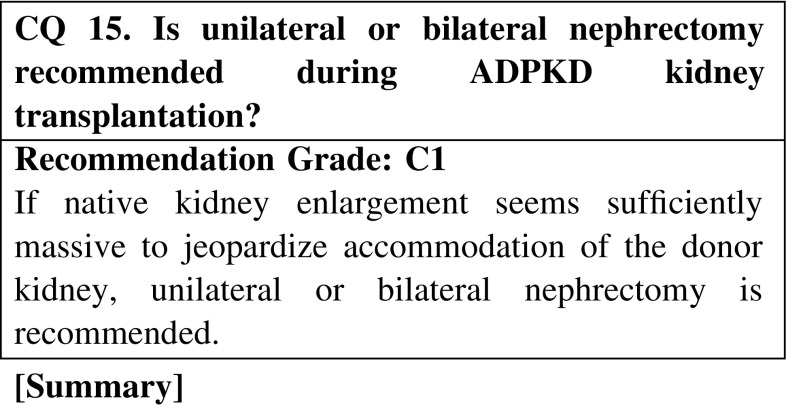


Renal transplantation for ADPKD patients proceeds routinely as it does for other patients, including incorporation of immunosuppressive therapy. Posttransplant survival is more favorable for ADPKD than for other ESRD patients. However, patients should be monitored postoperatively for possible complications such as thromboembolism, hyperlipidemia, postoperative diabetes onset, and hypertension. Careful screening is required to ensure that any kidney from a living donor is free of ADPKD. If the patient has a cerebral aneurysm, treatment is preferable prior to renal transplantation. If native kidney enlargement seems sufficiently massive to jeopardize accommodation of the donor kidney, unilateral (or rarely, bilateral) nephrectomy is recommended. However, there is no professional consensus on issues such as nephrectomy timing (simultaneous or heterochronic), scope (unilateral or bilateral), or method (open or laparoscopic).

## 5. Autosomal recessive polycystic kidney disease (ARPKD): disease concept/definition (etiology and pathophysiological mechanism)

ARPKD is a hereditary cystic kidney disease and inherited as an autosomal recessive trait. It is characterized by cystic dilation of renal collecting ducts and varying degrees of hepatic abnormalities consisting of biliary dysgenesis, and periportal fibrosis and bile duct proliferation in the liver. Generally, the hepatic lesion in ARPKD is clinically called congenital hepatic fibrosis if it presents alone, and is associated with the histological feature called ductal plate malformation. ARPKD is caused by mutations in *PKHD1*, located on chromosome 6p21.1-p12, and linkage analysis indicates that this disorder involves a single defective gene despite the wide variability in clinical presentation. It is found that causative gene proteins in three human PKDs (PKD1, PKD2, and ARPKD) are associated with primary cilia and the related structures, and it is inferred that structural abnormality and dysfunction of the primary cilia cause disease, and it is a theoretical rationale for the common pathophysiological mechanism of ARPKD and ADPKD.

## 6. ARPKD: diagnosis (symptomatology, symptom, and examination finding)

Renal ultrasonographic findings and a sibling with a history of ARPKD are important for the diagnosis of ARPKD. Cysts are usually small, and have mainly diffuse dilatations rather than a round shape. Renal ultrasonography demonstrates markedly enlarged echogenic kidneys, not a hubble-bubble low-echogenic appearance, and this recognition is important for diagnosis. Sonographic features of ARPKD may manifest in the second trimester but usually are not apparent until after 30 weeks’ gestation. Many diseases present with kidney cysts, all of which can be differential diagnoses. Among hereditary cystic kidney diseases, ADPKD is an important differential diagnosis. Occasionally, even in ARPKD, dilatation of the collecting ducts is not detected and macrocysts are present, which is a feature to notice. In advanced cases of ARPKD, it is sometimes difficult to morphologically distinguish ARPKD from ADPKD. Although ARPKD presents in infancy in most patients, a subset presents later in childhood and even adulthood, with abdominal distension related to renal enlargement or splenohepatomegaly.

## 7. ARPKD: epidemiology and prognosis (incidence, prevalence, and treatment outcome)

The incidence of ARPKD is inferred to be one case per 10,000–40,000 births. Prognosis is difficult to assess, although now it becomes clear that survival of all but the most severely affected neonates who demonstrate pulmonary hypoplasia is possible. It is expected that the prognosis will be improved in the future through improvement in the treatment of end-stage renal failure and disease management in infants early after birth.

## 8. ARPKD: prenatal diagnosis

In ARPKD, considering that patients often show severe clinical features early after birth, the prenatal diagnosis is useful in disease management. Prenatal diagnosis involves fetal ultrasonography and MRI, and there is no doubt of the clinical significance of performing these diagnostic imaging methods when required in present conditions of perinatal medical care. However, the precision of imaging techniques such as ultrasonography is low, and cysts of ARPKD are usually inapparent until 30 weeks’ gestation. Prenatal diagnosis of ARPKD by genetic analysis is established technically, and its enforcement is considered when a sibling is diagnosed with ARPKD. However, the request for a genetic examination from an overseas laboratory as an option may be subjected to genetic counseling because the enforcement of prenatal genetic diagnosis in Japan is difficult.

## 9. ARPKD: treatment and management of complications (treatment of disease including adjunct therapy, supportive therapy, and prophylaxis)


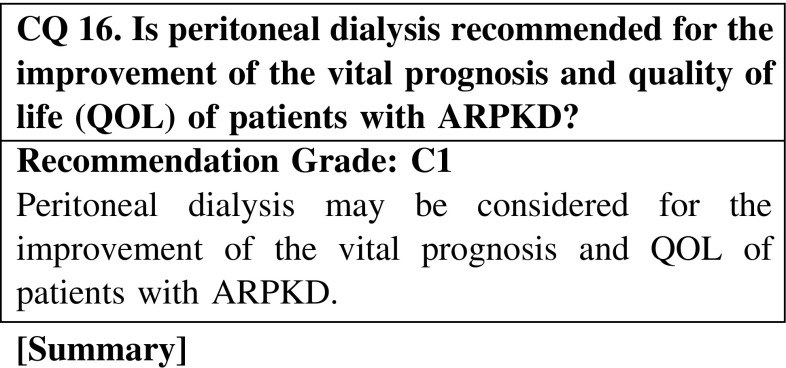


Peritoneal dialysis is considered for the improvement of the vital prognosis and QOL of patients with ARPKD. End-stage renal failure is often seen in ARPKD, and a replacement therapy for the kidney is required for those cases. Generally, hemodialysis is often unsuitable for children, and peritoneal dialysis is recommended when there are no special circumstances. It is a consensus that peritoneal dialysis is recommended for the improvement of the vital prognosis and QOL of patients with ARPKD considering the present conditions in the medical care of renal failure.
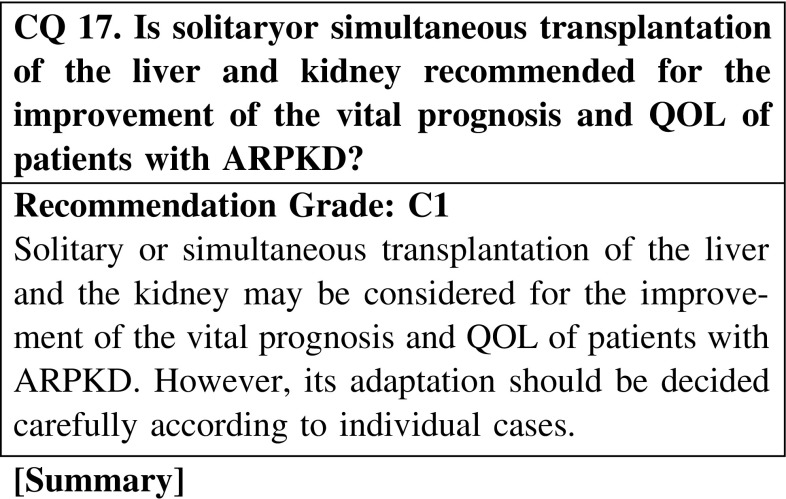


Although solitary or simultaneous transplantation of the liver and the kidney should be considered for the improvement of the vital prognosis and QOL of patients with ARPKD, its adaptation should be decided carefully according to individual cases. In ARPKD, because patients often show severe renal failure early after birth, a replacement therapy for the kidney is required. Generally, the best replacement therapy method for the kidney in children is thought to be renal transplantation, and its early enforcement is recommended. When the management of portal hypertension or recurrent bacterial cholangitis is difficult in the case of liver disorder in ARPKD patients, liver transplantation is considered. Although solitary or simultaneous transplantation of the liver and kidney should be considered for the improvement of the vital prognosis and QOL of patients with ARPKD considering the present conditions of transplantation medical care, its enforcement does not necessarily result in the improvement of vital prognosis and QOL in each case.
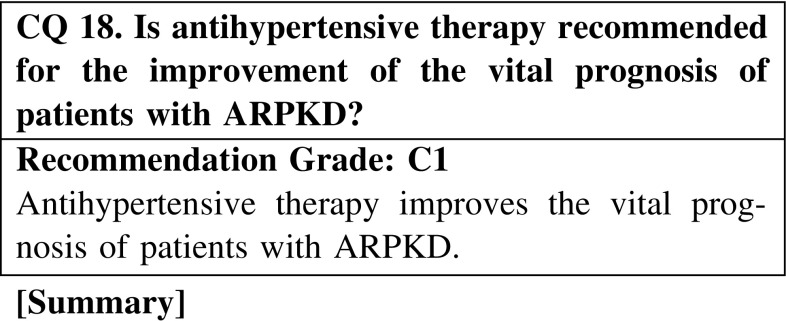


Antihypertensive therapy improves the vital prognosis of patients with ARPKD. Therefore, it may be considered a management option for ARPKD. Hypertension is often found in infants and subsequent childhood in ARPKD, and it can be the only symptom. Hypertension is also seen in patients with normal renal function and is manifested in almost all children with ARPKD. If hypertension is not treated effectively, hypercardia or congestive heart disorder may occur. The pathogenesis of hypertension in ARPKD is unknown. It is a consensus that antihypertensive therapy should be considered for the improvement of the vital prognosis of patients with ARPKD.

### **Acknowledgments**

Clinical Guidelines for Polycystic Kidney Disease 2014 Advisory Committee. Committee chairman: Shigeo Horie. Committee member: Toshio Mochizuki, Satoru Muto, Kazushige Hanaoka, Yoshimitsu Fukushima, Ichiei Narita, Kikuo Nutahara, Ken Tsuchiya, Kazuhiko Tsuruya, Koichi Kamura, Saori Nishio, Tatsuya Suwabe, Yoshifumi Ubara, Eiji Ishimura, Koichi Nakanishi. Collaborator: Keiichi Furukawa. Chief Chairman of the Clinical Practice Guidelines for Progressive Kidney Diseases: Kenjiro Kimura. Leader of the Research for Progressive Kidney Diseases of the Ministry of Health, Labour and Welfare: Seiichi Matsuo. Cooperative Medical Society: The Japanese Urological Association, The Japanese Society for Dialysis Therapy, The Japanese Society for Pediatric nephrology, The Japan Society of Human Genetics, The Japan Neurosurgical society, The Japanese Association for Infectious disease, The Japan Society of Hepatology, The Japanese Society of Interventional Radiology, The Japan Society for Transplantation.

**Bibliography**

I. Disease concept and definition of autosomal dominant polycystic kidney disease (ADPKD)

1. Grantham JJ. N Engl J Med. 2008;359:1477–85.
2. Reed B, et al. Am J Kidney Dis. 2010;56:50–6.
3. Grantham JJ, et al. Clin J Am Soc Nephrol. 2010;5:889–96.
4. Mekahli D, et al. Pediatr Nephrol. 2010;25:2275–82.

II. Diagnosis of ADPKD: symptoms and laboratory findings

1. Algorithm

1. Barua M, et al. Semin Nephrol. 2010;30:356–65.
2. Pei Y. Clin J Am Soc Nephrol. 2006;1:1108–14.
3. Pei Y, et al. Adv Chronic Kidney Dis. 2010;17:140–52.
4. Gabow PA. N Engl J Med. 1993;329:332–42.

2. Diagnostic criteria

1. Grantham JJ. N Engl J Med. 2008;359:1477–85.
2. Ravine D, et al. Am J Kidney Dis. 1993;22:803–7.
3. McHugh K, et al. Radiology. 1991;178:383–5.
4. Nascimento AB, et al. Radiology. 2001;221:628–32.
5. Belibi FA, et al. J Am Soc Nephrol. 2009;20:6–8.
6. Barua M, et al. Semin Nephrol. 2010;30:356–65.
7. Pei Y, et al. Adv Chronic Kidney Dis. 2010;17:140–52.
8. Chapman AB, et al. Semin Nephrol. 2011;31:237–44.
9. Pei Y. Clin J Am Soc Nephrol. 2006;1:1108–14.

3. Comparison of diagnostic criteria between Japan and other countries

1. Ravine D, et al. Am J Kidney Dis. 1993;22:803–7.
2. Ravine D, et al. Lancet. 1994;343:824–7.
3. Pei Y, et al. J Am Soc Nephrol. 2009;20:205–12.
4. Bear JC, et al. Am J Med Genet. 1984;18:45–53.
5. Belibi FA, et al. J Am Soc Nephrol. 2009;20:6–8.
6. Barua M, et al. Semin Nephrol. 2010;30:356–65.
7. Pei Y, et al. Adv Chronic Kidney Dis. 2010;17:140–52.
8. Pei Y. Clin J Am Soc Nephrol. 2006;1:1108–14.
9. Harris PC, et al. J Am Soc Nephrol. 2006;17:3013–9.

4. Testing

1. Matsuo S, et al. Am J Kidney Dis. 2009;53:982–92.
2. Pei Y. Clin J Am Soc Nephrol. 2006;1:1108–14.

5. Diagnostic imaging

1. Barua M, et al. Semin Nephrol. 2010;30:356–65.
2. Pei Y, et al. Adv Chronic Kidney Dis. 2010;17:140–52.
3. Pei Y. Clin J Am Soc Nephrol. 2006;1:1108–14.
4. Cadnapaphornchai MA, et al. Clin J Am Soc Nephrol. 2009;4:820–9.
5. Nascimento AB, et al. Radiology. 2001;221:628–32.
6. Wolyniec W, et al. Pol Arch Med Wewn. 2008;118:767–73.
7. Bae KT, et al. J Comput Assist Tomogr. 2000;24:614–9.
8. Grantham JJ. N Engl J Med. 2008;359:1477–85.
9. Grantham JJ, et al. Clin J Am Soc Nephrol. 2006;1:148–57.
10. Grantham JJ, et al. N Engl J Med. 2006;354:2122–30.
11. Ramunni A, et al. Hypertens Res. 2004;27:221–5.
12. Kondo A, et al. Int J Urol. 2001;8:95–8.
13. King BF, et al. Kidney Int. 2003;64:2214–21.
14. Pirson Y, et al. J Am Soc Nephrol. 2002;13:269–76.
15. Vega C, et al. Am Fam Phys. 2002;15:601–8.
16. Satoh T. No Shinkei Geka. 2002;30:487–93 (Japanese).
17. Gieteling EW, et al. J Neurol. 2003;250:418–23.
18. Ross JS, et al. Am J Neuroradiol. 1990;11:449–55.
19. Johnson AM, et al. J Am Soc Nephrol. 1997;8:1560–7.
20. Gabow PA, et al. Kidney Int. 1992;41:1311–9.
21. Torra R, et al. J Am Soc Nephrol. 1996;7:2142–51.
22. Bear JC, et al. Am J Med Genet. 1984;18:45–53.

6. Differential diagnosis

1. Barua M, et al. Semin Nephrol. 2010;30:356–65.
2. Pei Y. Clin J Am Soc Nephrol. 2006;1:1108–14.
3. Wolyniec W, et al. Pol Arch Med Wewn. 2008;118:767–73.
4. Brook-Carter PT, et al. Nat Genet. 1994:8;328–32.
5. Sampson J, et al. Am J Hum Genet. 1997;61:843–51.
6. Fick GM, et al. J Am Soc Nephrol. 1993;3:1863–70.
7. Hoevenaren IA, et al. Liver Int. 2008;28:264–70.
8. Li A, et al. Am J Hum Genet. 2003;72:691–703.
9. Davila S, et al. Nat Genet. 2004;36:575–7.
10. Drenth JP, et al. Nat Genet. 2003;33:345–7.
11. Pei Y, et al. Adv Chronic Kidney Dis. 2010;17:140–52.
12. Calvet JP. Clin J Am Soc Nephrol. 2008;3:1205–11.
13. Torres VE, et al. Lancet. 2007;369:1287–301.

7. Genetic diagnosis

1. The European Polycystic Kidney Disease Consortium. Cell. 1994;77:881–94.
2. Mochizuki T, et al. Science. 1996;272:1339–42.
3. Harris P, et al. Nature Rev Nephrol. 2010;6:197–206.
4. Huang E, et al. Transplantation. 2009;87:133–7.
5. Brun M, et al. Ultrasound Obstet Gynecol. 2004;24:55–61.

8. Diagnostic imaging for infants and young adults

1. Cadnapaphornchai MA, et al. Kidney Int. 2008;74:1192–6.
2. Schrier RW, et al. J Am Soc Nephrol. 2004;15:1023–8.

9. Initial symptoms

1. Grantham JJ. N Engl J Med. 2008;359:1477–85.
2. Hogan MC, et al. Adv Chronic Kidney Dis. 2010;17:e1–16.
3. Bajwa ZH, et al. Kidney Int. 2004;66:1561–9.
4. Gabow PA, et al. Am J Kidney Dis. 1992;20:140–3.
5. Johnson AM, et al. J Am Soc Nephrol. 1997;8:1560–7.
6. Ubara Y, et al. Am J Kidney Dis. 1999;34:926–31.

10. Renal symptoms

1. Gabow PA. N Engl J Med. 1993;329:332–42.
2. Bajwa ZH, et al. Kidney Int. 2004;66:1561–9.
3. Elzinga LW, et al. J Am Soc Nephrol. 1992;2:1219–26.
4. Elzinga LW, et al. Am J Kidney Dis. 1993;22:532–7.
5. Contreras G, et al. J Am Soc Nephrol. 1995;6:1354–9.
6. Seeman T, et al. Physiol Res. 2004;53:629–34.
7. Torres VE. Kidney Int. 2005;68:2405–18.
8. Grantham JJ, et al. Clin J Am Soc Nephrol. 2006;1:148–57.
9. Torres VE, et al. Kidney Int. 2009;76:149–68.
10. Johnson AM, et al. J Am Soc Nephrol. 1997;8:1560–7.
11. Grantham JJ, et al. N Engl J Med. 2006;354:2122–30.
12. Gabow PA, et al. Kidney Int. 1992;41:1311–9.
13. Peters DJ, et al. Lancet. 2001;358:1439–44.
14. Fick-Brosnahan GM, et al. Am J Kidney Dis. 2002;39:1127–34.
15. Rossetti S, et al. J Am Soc Nephrol. 2007;18:1374–80.
16. Tokiwa S, et al. Clin Exp Nephrol. 2011;15:539–45.
17. Higashihara E, et al. Clin Exp Nephrol. 2012;16:622–8.

III. ADPKD: epidemiology and prognosis (prevalence, incidence, renal prognosis, and vital prognosis)

1. Higashihara E, et al. Nephron. 1998;80:421–7.
2. Davies F, et al. Q J Med. 1991;79:477–85.
3. de Almeida E, et al. Kidney Int. 2001;59:2374.
4. Higashihara E, et al. All of polycystic kidney disease. Inter Medica, Tokyo, Japan. 2006;16–21 (Japanese).
5. Iglesias CG, et al. Am J Kidney Dis. 1983;2:630–9.
6. Flick GM, et al. J Am Soc Nephrol. 1995;5:2048–56.
7. Higashihara E, et al. All of polycystic kidney disease. Inter Medica, Tokyo, Japan. 2006;225–232 (Japanese).
8. Perrone RD, et al. Am J Kidney Dis. 2001;38:777–84.

IV. ADPKD: treatment and management of complications

1. Treatment to control the development of ADPKD

(1) Antihypertensive treatment

CQ 1. Is antihypertensive treatment recommended as a means of slowing the deterioration of renal function in patients with ADPKD complicated with hypertension?

1. Cadnapaphornchai MA, et al. Clin J Am Soc Nephrol. 2009;4:820–9 (Level 2).
2. Schrier RW, et al. Kidney Int. 2003;63:678–85 (Level 2).
3. Jafar TH, et al. Kidney Int. 2005;67:265–71 (Level 1).
4. Maschio G, et al. N Engl J Med. 1996;334:939–45 (Level 2).
5. van Dijk MA, et al. Nephrol Dial Transplant. 2003;18:2314–20 (Level 2).
6. Ecder T, et al. Am J Kidney Dis. 2000;35:427–32 (Level 2).
7. Schrier R, et al. J Am Soc Nephrol. 2002;13:1733–9 (Level 2).
8. Kanno Y, et al. QJM. 1996;89:65–70 (Level 4).
9. Nutahara K, et al. Nephron Clin Pract. 2005;99:c18–23 (Level 2).
10. Mitobe M, et al. Clin Exp Nephrol. 2010;14:573–7 (Level 4).
11. Zeltner R, et al. Nephrol Dial Transplant. 2008;23:573–9 (Level 2).
12. Ecder T, et al. Am J Nephrol. 2001;21:98–103 (Level 3).
13. Sarnak MJ, et al. Ann Intern Med. 2005;142:342–51 (Level 2).

(2) Increased water intake

CQ 2. Does increased water intake have a beneficial effect in ADPKD patients?

1. Wang X, et al. J Am Soc Nephrol. 2008;19:102–8 (Level 4).
2. Nagao S, et al. J Am Soc Nephrol. 2006;17:2220–7 (Level 4).
3. Gabow PA, et al. Kidney Int. 1989;35:675–80 (Level 3).
4. Zittema D, et al. Clin J Am Soc Nephrol. 2012;7:906–13 (Level 3).
5. Ho TA, et al. Kidney Int. 2012;82:1121–9 (Level 3).
6. Torres VE, et al. Clin J Am Soc Nephrol. 2009;4:1140–50 (Level 6).
7. Barash I, et al. Clin J Am Soc Nephrol. 2010;5:693–7 (Level 4).
8. Wang CJ, et al. Clin J Am Soc Nephrol. 2011;6:192–7 (Level 4).

(3) Dietary protein restriction

CQ 3. Should we recommend dietary protein restriction to inhibit progression of renal dysfunction in patients with ADPKD?

1. Locatelli F, et al. Lancet. 1991;337:1299–304 (Level 2).
2. Choukroun G, et al. J Am Soc Nephrol. 1995;6:1634–42 (Level 4).
3. Klahr S, et al. J Am Soc Nephrol. 1995;5:2037–47 (Level 2).
4. Levey AS, et al. Am J Kidney Dis. 2006;48:879–88 (Level 2).
5. Oldrizzi L, et al. Kidney Int. 1985;27:553–7 (Level 3).
6. Fouque D, et al. Cochrane Database Syst Rev. 2009;3:CD001892 (Level 1).

(4) Tolvaptan

CQ 4. Is tolvaptan recommended for treatment of ADPKD?

1. Torres VE, et al. N Engl J Med. 2012;367:2407–18 (Level 2).
2. Higashihara E, et al. Clin J Am Soc Nephrol. 2011;2499–507 (Level 3).
3. Irazabal MV, et al. Kidney Int. 2011;80:295–301 (Level 4).

(5) Aspiration of renal cysts

CQ 5. Aspiration of renal cysts in patients with ADPKD

1. Skolarikos A, et al. BJU Int. 2012;110:170–8 (Level 4).
2. Millar MB, et al. J Endourol. 2013;27:528–34 (Level 4).
3. Higashihara E, et al. J Urol. 1992;147:1482–4 (Level 4).
4. Bennett WM, et al. J Urol. 1987;137:620–2 (Level 4).
5. Uemasu J, et al. Nephrol Dial Transplant. 1996;11:843–6 (Level 4).
6. Kim SH, et al. Korean J Radiol. 2009;10:377–83 (Level 4).
7. Lee YR, et al. Korean J Radiol. 2003;4:239–42 (Level 4).
8. Chapman AB, et al. Am J Kidney Dis. 1990;16:252–5 (Level 5).
9. Gupta S, et al. Acta Radiol. 2000;41:280–4 (Level 4).
10. Fleming TW, et al. J Urol. 1998;159:44–7 (Level 4).

2. Complications and their managements

(1) Cerebral aneurysm and subarachnoid hemorrhage

CQ 6:Does screening of intracranial aneurysms improve the prognosis of ADPKD patients?

1. Chauveau D, et al. Kidney Int. 1994;45:1140–6 (Level 4).
2. Schievink WI, et al. J Am Soc Nephrol. 1992;3:88–95 (Level 4).
3. Vlak MH, et al. Lancet Neurol. 2011;10:626–36 (Level 4).
4. Irazabal MV, et al. Clin J Am Soc Nephrol. 2011;6:1274–85 (Level 4).
5. Xu HW, et al. Stroke. 2011;42:204–6 (Level 4).
6. Gieteling EW, et al. J Neurol. 2006;250:418–23 (Level 4).
7. Morita A, et al. N Engl J Med. 2012;366:2474–82 (Level 4).

CQ 7. Is treatment recommended for cerebral aneurysms detected during screening?

1. Rinkel GJ. J Neuroradiol. 2008;35:99–103 (Level 6).
2. Hughes PD, et al. Nephrology (Carlton). 2003;8:163–70 (Level 6).

(2) Cyst infection

CQ 8. Are newer quinolones recommended for the treatment of cyst infection in ADPKD?

1. Alam A, et al. Clin J Am Soc Nephrol. 2009;4:1154–5 (Level 6).
2. Sallée M, et al. Clin J Am Soc Nephrol. 2009;4:1183–9 (Level 4).
3. Suwabe T, et al. Nephron Clin Pract. 2009;112:157–63 (Level 4).
4. Schwab SJ, et al. Am J Med. 1987;82:714–18 (Level 4).
5. Muther RS, et al. Kidney Int. 1981;20:519–22 (Level 5).
6. Bennet WM, et al. Am J Kidney Dis. 1985;6:400–4 (Level 5).
7. Schwab SJ, et al. Am J Kidney Dis. 1983;3:63–6 (Level 5).
8. Elzinga LW, et al. Kidney Int. 1987;32:884–8 (Level 5).
9. Elzinga LW, et al. Antimicrob Agents Chemother. 1988;32:844–7 (Level 5).
10. Telenti A, et al. Mayo Clin Proc. 1990;65:933–42 (Level 5).
11. Rossi SJ, et al. Ann Pharmacother. 1993;27:38–9 (Level 5).
12. Hiyama L, et al. Am J Kidney Dis. 2006;47:E9–13 (Level 5).

(3) Cystic hemorrhage/hematuria

CQ 9. Should we recommend tranexamic acid in the treatment of cystic hemorrhage in ADPKD?

1. Johnson AM, et al. J Am Soc Nephrol. 1997;8:1560–7 (Level 4).
2. Peces R, et al. Nefrologia. 2012;32:160–5 (Level 5).
3. Vujkovac B, et al. Blood Coagul Fibrinolysis. 2006;17:589–91 (Level 5).
4. Alameel T, et al. J Int Nephrol. 2011;203579 (Level 5).

(4) Urolithiasis

CQ 10. Are there any effective pharmacological preventive therapies for urolithiasis associated with ADPKD?

1. Higashihara E, et al. J Urol. 1992;147:329–32 (Level 4).
2. Grampsas SA, et al. Am J Kidney Dis. 2000;36:53–7 (Level 4).
3. Nishiura JL, et al. Clin J Am Soc Nephrol. 2009;4:838–44 (Level 4).
4. Torres VE, et al. Am J Kidney Dis. 1988;11:318–25 (Level 4).

(5) Cardiac complications (including valvular disease)

CQ 11. Is transthoracic echocardiography (TTE) for screening of valvular disease recommended to improve the mortality of ADPKD patients?

1. Ecder T, et al. Nat Rev Nephrol. 2009;5:221–8 (Level 6).
2. Koren MJ, et al. Ann Intern Med. 1991;114:345–52 (Level 3).
3. Gabow PA, et al. Kidney Int. 1992;41:1311–9 (Level 4).
4. Chapman AB. J Am Soc Nephrol. 1997;8:1292–7 (Level 3).
5. Hossack KF, et al. N Engl J Med. 1988;319:907–12 (Level 3).
6. Flack JM, et al. Am Heart J. 1999;138:486–92 (Level 3).
7. Freed LA, et al. N Engl J Med. 1999;341:1–7 (Level 3).
8. Avierinos JF, et al. Circulation. 2002;106:1355–61 (Level 3).
9. Enriquez-Sarano M, et al. N Engl J Med. 2005;352:875–83 (Level 4).
10. Bonow RO, et al. J Am Coll Cardiol. 2006;48:e1–e148 (Level 1).
11. Zoghbi WA, et al. J Am Soc Echocardiogr. 2003;16:777–802 (Level 6).
12. Lumiaho A, et al. Am J Kidney Dis. 2001;38:1208–16 (Level 3).
13. Adeola T, et al. J Natl Med Assoc. 2001;93:282–7 (Level 5).
14. Hadimeri H, et al. J Am Soc Nephrol. 1998;9:837–41 (Level 5).
15. Ecder T, et al. Nephrol Dial Transplant. 1999;14:1113–6 (Level 4).

(6) The specific treatment of complications

CQ 12. Should ADPKD patients with ESRD undergo renal transarterial embolization to reduce enlarged kidneys?

1. Harley JD, et al. AJR Am J Roentgenol. 1980;134:818–20 (Level 5).
2. Hahn ST, et al. Cardiovasc Intervent Radiol. 1999;22:422–4 (Level 5).
3. Ubara Y, et al. Am J Kidney Dis. 1999;34:926–31 (Level 5).
4. Ubara Y, et al. Am J Kidney Dis. 2002;39:571–9 (Level 5).
5. Sakuhara Y, et al. J Vasc Interv Radiol. 2008;19:267–71 (Level 5).
6. Rim H, et al. Korean J Radiol. 2010;11:574–8 (Level 5).
7. Morishita H, et al. J Vasc Interv Radiol. 2011;22:1631–3 (Level 5).
8. Mukai T, et al. Acta Med Okayama. 2011;65:347–51 (Level 5).

CQ 13. Should ADPKD patients with ESRD undergo hepatic transarterial embolization to reduce hepatomegaly?

1. Ubara Y, et al. Am J Kidney Dis. 2004;43:733–8 (Level 5).
2. Takei R, et al. Am J Kidney Dis. 2007;49:744–52 (Level 5).
3. Park HC, et al. J Korean Med Sci. 2009;24:57–61 (Level 5).
4. Wang MQ, et al. Abdom Imag. 2013;38:465–73 (Level 5).

3. The treatment of ESRD

(1) Peritoneal dialysis

CQ 14. Is peritoneal dialysis recommended for patients with ADPKD?

1. Covic A, et al. Nephrol Dial Transpl. 2010;25:1757–9 (Level 4).
2. Fenton SS, et al. Am J Kidney Dis. 1997;30:334–42 (Level 3).
3. Schaubel DE, et al. Perit Dial Int. 1998;18:478–84 (Level 3).
4. Collins AJ, et al. Am J Kidney Dis. 1999;34:1065–74 (Level 3).
5. Heaf JG, et al. Nephrol Dial Transpl. 2002;17:112–7 (Level 3).
6. Moist LM, et al. J Am Soc Nephrol. 2000;11:556–64 (Level 3).
7. Foley RN, et al. J Am Soc Nephrol. 1998;9:267–76 (Level 3).
8. Vonesh EF, et al. Kidney Int. 2004;66:2389–401 (Level 3).
9. Liem YS, et al. Kidney Int. 2007;71:153–8 (Level 3).
10. Stack AG, et al. Kidney Int. 2003;64:1071–9 (Level 3).
11. Ganesh SK, et al. J Am Soc Nephrol. 2003;14:415–24 (Level 3).

(2) Renal transplantation

CQ 15. Is unilateral or bilateral nephrectomy recommended during ADPKD kidney transplantation?

1. Jacquet A, et al. Transpl Int. 2011;24:582–7 (Level 4).
2. Kramer A, et al. J Urol. 2009;181:724–8 (Level 4).
3. Sulikowski T, et al. Transplant Proc. 2009;41:177–80 (Level 4).
4. Hadimeri H, et al. Nephrol Dial Transpl. 1997;12:1431–6 (Level 4).
5. Fuller TF, et al. J Urol. 2005;174:2284–8 (Level 4).
6. Patel P, et al. Ann R Coll Surg Engl 2011;93:391—5. (Level 4)
7. Cohen D, et al. Prog Urol. 2008;18:642–9 (Level 4).
8. Yamamoto T, et al. Transplantation. 2012;93:794–8 (Level 4).
9. Desai MR, et al. BJU Int. 2008;101:94–7 (Level 4).
10. Gill IS, et al. J Urol. 2001;165:1093–8 (Level 4).
11. Jenkins MA, et al. Urology. 2002;59:32–6 (Level 4).
12. Dunn MD, et al. Am J Kidney Dis. 2000;35:720–5 (Level 4).
13. Bendavid Y, et al. Surg Endosc. 2004;18:751–4 (Level 4).

V. Autosomal recessive polycystic kidney disease (ARPKD): disease concept/definition (etiology and pathophysiological mechanism)

1. Ward CJ, et al. Nat Genet. 2002;30:259–69.
2. Onuchic LF, et al. Am J Hum Genet. 2002;70:1305–17.

VI. ARPKD: diagnosis (symptomatology, symptom, and examination findings)

1. Guay-Woodford LM, et al. Pediatrics. 2003;111:1072–80.
2. Capisonda R, et al. Pediatr Nephrol. 2003;18:119–26.
3. Zerres K, et al. Acta Paediatr. 1996;85:437–45.
4. Kääriäinen H, et al. Pediatr Radiol. 1988;18:45–50.
5. Gagnadoux MF, et al. Adv Nephrol Necker Hosp. 1989;18:33–57.
6. Roy S, et al. Pediatr Nephrol. 1997;11:302–6.

VII. ARPKD: epidemiology and prognosis (incidence, prevalence, and treatment outcome)

1. Zerres K, et al. Am J Med Genet. 1998;76:137–44.
2. Guay-Woodford LM, et al. Pediatrics. 2003;111:1072–80.
3. Bergmann C, et al. Kidney Int. 2005;67:829–48.

1. ARPKD: Prenatal diagnosis

1. Zerres K, et al. Am J Med Genet. 1998;76:137–44.
2. Zerres K, et al. Clin Genet. 2004;66:53–7.
3. Guay-Woodford LM, et al. Am J Hum Genet. 1995;56:1101–7.
4. Gigarel N, et al. Reprod Biomed Online. 2008;16:152–8.

2. ARPKD: treatment and management of complications (treatment of disease including adjunct therapy, supportive therapy, and prophylaxis)

CQ 16. Is peritoneal dialysis recommended for the improvement of the vital prognosis and quality of life (QOL) of patients with ARPKD?

1. Bergmann C, et al. Kidney Int. 2005;67:829–48 (Level 5).
2. Beaunoyer M, et al. Pediatr Transpl. 2007;11:267–71 (Level 5).
3. Spechtenhauser B, et al. Pediatr Transpl. 1999;3:246–8 (Level 5).

CQ 17. Is independent or simultaneous transplantation of the liver and kidney recommended for the improvement of the vital prognosis and QOL of patients with ARPKD?

1. Bergmann C, et al. Kidney Int. 2005;67:829–48 (Level 5).
2. Beaunoyer M, et al. Pediatr Transpl. 2007;11:267–71 (Level 5).
3. Spechtenhauser B, et al. Pediatr Transpl. 1999;3:246–8 (Level 5).
4. Gunay-Aygun M, et al. Clin J Am Soc Nephrol. 2010;5:972–84 (Level 5).
5. De Kerckhove L, et al. Transpl Int. 2006;19:381–8 (Level 5).
6. Davis ID, et al. Pediatr Transpl. 2003;7:364–9 (Level 5).
7. Khan K, et al. Am J Transpl. 2002;2:360–5 (Level 5).

CQ 18. Is antihypertensive therapy recommended for the improvement of the vital prognosis of patients with ARPKD?

1. Bergmann C, et al. Kidney Int. 2005;67:829–48 (Level 5).
2. Loghman-Adham M, et al. J Histochem Cytochem. 2005;53:979–88 (Level 5).

